# Seroprevalence of *Toxoplasma gondii* and *Borrelia burgdorferi* infections in patients with multiple sclerosis in Poland

**DOI:** 10.1038/s41598-024-61714-y

**Published:** 2024-05-14

**Authors:** Agnieszka Pawełczyk, Katarzyna Donskow-Łysoniewska, Ludmiła Szewczak, Magdalena Kierasińska, Maja Machcińska, Rafał Rola, Renata Welc-Falęciak

**Affiliations:** 1 Department of Immunopathology of Infectious and Parasitic Diseases, Medical University of Warsaw, 3C Pawińskiego Street, 02-106 Warsaw, Poland; 2 Laboratory of Parasitology, General Karol Kaczkowski Military Institute of Hygiene and Epidemiology, Kozielska 4 Street, 01-163 Warsaw, Poland; 3 Department of Experimental Immunotherapy, Faculty of Medicine, Lazarski University, 43 Świeradowska Street, 02-662 Warsaw, Poland; 4 Department of Parasitology, Faculty of Biology, University of Warsaw, 1 Miecznikowa Street, 02-096 Warsaw, Poland; 5 Department of Neurology, Military Institute of Aviation Medicine, Krasińskiego 54/56 Street, 01-755 Warsaw, Poland

**Keywords:** Multiple sclerosis, Toxoplasmosis, Lyme disease, Lyme borreliosis, *Toxoplasma*, *Borrelia*, Multiple sclerosis, Parasitic infection, Bacterial infection, Epidemiology

## Abstract

Multiple sclerosis (MS) is a chronic, demyelinating disease of the central nervous system that affects mainly young people. It is believed that the autoimmune process observed in the pathogenesis of MS is influenced by a complex interaction between genetic and environmental factors, including infectious agents. The results of this study suggest the protective role of *Toxoplasma gondii* infections in MS. Interestingly, high *Toxoplasma* IgM seropositivity in MS patients receiving immunomodulatory drugs (IMDs) was identified. On the other hand, *Borrelia* infections seem to be positively associated with MS. Although the interpretation of our results is limited by the retrospective nature of the studies, the results strongly indicate that further experimental and clinical studies are needed to explain the role of infectious agents in the development and pathophysiological mechanisms of MS.

## Introduction

Multiple sclerosis (MS) is a chronic, demyelinating disease of the central nervous system (CNS) that affects mainly young people. At the time of diagnosis, 85% of MS suffers had Relapsing–remitting MS (RRMS). Of the remainder, 10% were diagnosed with Primary-progressive MS (PPMS) and 5% with Progressive-relapsing MS (PRMS). It is believed that the autoimmune process observed in the pathogenesis of MS is influenced by a complex interaction between genetic and environmental factors ^1–3^. Several infectious agents, i.e. the Epstein-Barr virus (EBV), have been associated with the increasing risk of future development of MS ^3,4^. On the other hand, parasitic infections (helminths) have been shown to play an important role in modulating the immune system of the host and to reduce the risk of autoimmune and allergic diseases according to the hygiene hypothesis ^3,5^.

*Toxoplasma gondii* is an intracellular parasite that causes toxoplasmosis in humans and animals worldwide. The global seroprevalence of *T. gondii* infection is estimated at about 30% and varies depending on the geographical region, from 1% to over 90% ^6^. Low seroprevalences (10 to 30%) have been observed in North America, in South East Asia, in Northern Europe, and in Sahelian countries of Africa ^6^. Moderate prevalences (30 to 50%) have been found in countries of Central and Southern Europe, and high prevalences have been found in Latin America and in tropical African countries ^6^. It is worth noting that highest MS rates are recorded in Northern Europe, the British Isles and Scandinavia in particular, where low seroprevalence of *Toxoplasma* is observed ^6,7^. Whereas in immunocompetent individuals, *Toxoplasma* infection is usually asymptomatic, in immunocompromised patients (i.e. HIV-infected individuals, transplant recipients) may result in life-threatening toxoplasmic encephalitis as well as pulmonary or disseminated toxoplasmosis ^6^. So far, the relationship between *T. gondii* infection and autoimmune diseases including Parkinson’s ^8^, Alzheimer’s disease ^9^, rheumatoid arthritis ^10^, as well as psychiatric diseases (i.e. depression, bipolar disorder, schizophrenia; ^11,12^), have been observed. Nevertheless, whether the *T. gondii* infection is associated with MS seems to be controversial and the results of published papers are inconsistent. Recently studies and meta-analysis have suggested protective effect of *Toxoplasma* infection on the risk of being diagnosed with MS supporting the hygiene hypothesis ^13–16^. On the other hand, Oruç and co-authors ^17^ have shown that *Toxoplasma* IgG seropositivity was positively associated with MS. A recent meta-analysis has shown lower prevalence of *T. gondii* in MS patients, compared to control groups; however, no significant associations were reported ^18^. Although Shapira et al. reported that *T. gondii* IgG was higher in autoimmune diseases, no association was found between *T. gondii* IgG seropositivity and MS in two different geographical regions ^19^.

With 85,000 cases reported annually, Lyme borreliosis (LB) is the most common vector-borne disease in Europe ^20^. Bacteria, transmitted by ticks, can spread from the skin to other tissues and organs, including central and peripheral nervous system, and cause Lyme neuroborreliosis (LNB; 21). The clinical manifestations of LNB can overlap with other autoimmune, neurodegenerative diseases such as MS ^22,23^. The *Borrelia* infections were suggested as a cause of MS since the correlation between the clinically confirmed diagnosis of MS and the positive results of serological tests with *Borrelia* antigens have been observed ^24–26^. On the contrary, Forrester and co-authors ^27^ have found no geographic correlation between Lyme borreliosis and deaths due to neurodegenerative disorders, including MS.

It is estimated that in 2020, approximately 2,800,000 people worldwide were affected by multiple sclerosis ^28^. There is a noticeable and consistent increase in the number of affected individuals. According to the national report of the Polish Ministry of Health (https://ezdrowie.gov.pl; ^29^), the number of patients with MS has fluctuated between 43,000 and 44,000 in recent years in Poland.

To our knowledge, only one study about *Borrelia* seroprevalence in MS patients was conducted in Poland ^30^. The authors have concluded that MS may be often associated with *Borrelia* infection, however, the relatively low number of definite MS cases included in the study suggests that the results should be interpreted with caution. No studies about the seroprevalence of *T. gondii* in MS patients in Poland have been conducted until now. The aim of our study was to investigate the seroprevalence of *T. gondii* and *B. burgdorferi* s.l. in the Polish population of MS patients.

## Materials and methods

### Study design and human subjects

The retrospective study was conducted on adult patients (> 18 years) with clinically definite relapsing–remitting (RRMS; n = 122) or primary-progressive (PPMS; n = 2) MS who were admitted to the Military Institute of Aviation Medicine in Warsaw or the Department of Neurology in the Medical University of Warsaw in 2017–2022 (n = 124). The RRMS clinical form was determined according to the classification of Lublin and Reingold ^31^. The clinical disability was evaluated using the Kurtzke Expanded Disability Status Scale (EDSS) at the time of patient enrolment (0.0–6.0). The lesion location (brain, spinal cord and optic nerve) and the results of Gadolinium (Gd) contrast-enhancing lesions were obtained from the MRI scan. All patients with RRMS included to the study were in remission (n = 122; Supplementary File 1). More women than men participated in this study (83/121; 68.6% vs. 38/121; 31.4%; not data about sex n = 3) and the mean age of participants was 38 years (range 19–58; not data about age n = 3). Of the 124 MS patients, 91 (73.4%) were receiving immunomodulatory drugs (IMDs) and were treated with interferon beta (n = 28; 30.8%), glatiramer acetate (n = 26; 28.6%), dimethyl fumarate (n = 15; 16.5%), natalizumab (n = 15; 16.5%), teriflunomide (n = 6; 6.6%) or fingolimod (n = 1; 1.1%) (Supplementary File 1). All treated patients were receiving the same IMD at least for 3 months.

The control group was represented by 150 healthy blood donors from whom serum samples were collected in 2016. More men than women participated in this study (88/150; 58.6% vs. 62/150; 41.3%) and the mean age of participants was 34 years (range 18–71; not data about age n = 1). Blood samples were collected by antecubital venipuncture. Sera obtained from MS patients and healthy donors were frozen at − 80 °C until further analysis.

### Inclusion and exclusion criteria

All MS patients have to meet the 2010 Revised McDonald Criteria for a diagnosis of MS. Only patients who did not have a history of malignancies, or other autoimmune or neurodegenerative diseases, had no acute or chronic systemic infections (bacterial, viral or fungal), diagnosis of AIDS as well as did not receive any corticosteroid (within 30 days prior) or antiparasitic treatment were included to the study. Women in pregnant and patients > 65 years were excluded from the study.

The control group consisted of unrelated healthy blood donors who were from the same geographical area as the patients. Individuals with immunodeficiency or who received IMDs were excluded for further study.

### Serological tests

All serum samples (124 from MS patients and 150 from blood-healthy donors) were analysed for the presence of antibodies against *T. gondii* and *B. burgdorferi* s.l. by using commercially available tests for in vitro diagnostic (IVD) with the manufacturer’s interpretation criteria (*Toxoplasma gondii* IgM and IgG ELISA, *Borrelia* 14 kDa OspC IgM ELISA, *Borrelia* IgG + VIsE ELISA, DRG Instruments GmbH, Marburg, Germany). *Toxoplasma* IgG antibody avidity was determined only for IgG-positive samples (*Toxoplasma gondii* IgG Avidity ELISA, DRG Instruments GmbH, Marburg, Germany). For confirmation of positive or doubtful results of *Borrelia* ELISA test, Western Blot test (Anti-*Borrelia* IgM and IgG Line Immunoassay, DRG Instruments GmbH, Marburg, Germany) was performed according to the recommendations of The Polish Society of Epidemiology and Infectious Disease ^32^ and European guidelines ^33^.

### Statistical analysis

Statistical analysis was performed using IBM SPSS Statistics v. 28.0 software (SPSS IBM Corp., Armonk, NY, USA). For the analysis of the results, doubtful serological results of tested pathogens were classified as negative. Seroprevalence rates were compared with the tested group (MS patients/blood donors) using Maximum Likelihood techniques based on log-linear analysis of contingency tables. For analysis of the IgM and IgG seroprevalence of *Borrelia* and *Toxoplasma* pathogens, we fitted the seroprevalence of each pathogen as a binary factor in each immunoglobulin class (infected = 1, uninfected = 0) and then by tested groups (2 levels: MS patients and blood donors), age (4 levels: < 20 years, 21–35 years, 36–50 years and > 51), sex (2 levels: female and male) and IMDs treatment (2 levels: yes and no; only for group of MS patients). For each level of analysis, beginning with the most complex model, involving all possible main effects and interactions, those combinations not contributing significantly to the explanation of variation in the data were eliminated stepwise, beginning with the highest-level interaction. A minimum sufficient model was then obtained, for which the likelihood ratio of χ2 was not significant, indicating that the model was sufficient in explaining the data. P values < 0.05 were considered to be statistically significant.

### Ethics declarations

The Internal Review Board of the Warsaw Medical University was informed about the study protocol (no. AKBE/103/2022). The study protocol followed ethical guidelines of the 2013 Declaration of Helsinki. All ethical approvals for the study have been obtained according to Polish regulations.

## Results

### Toxoplasma gondii IgM and IgG seroprevalence

**Figure 1:**
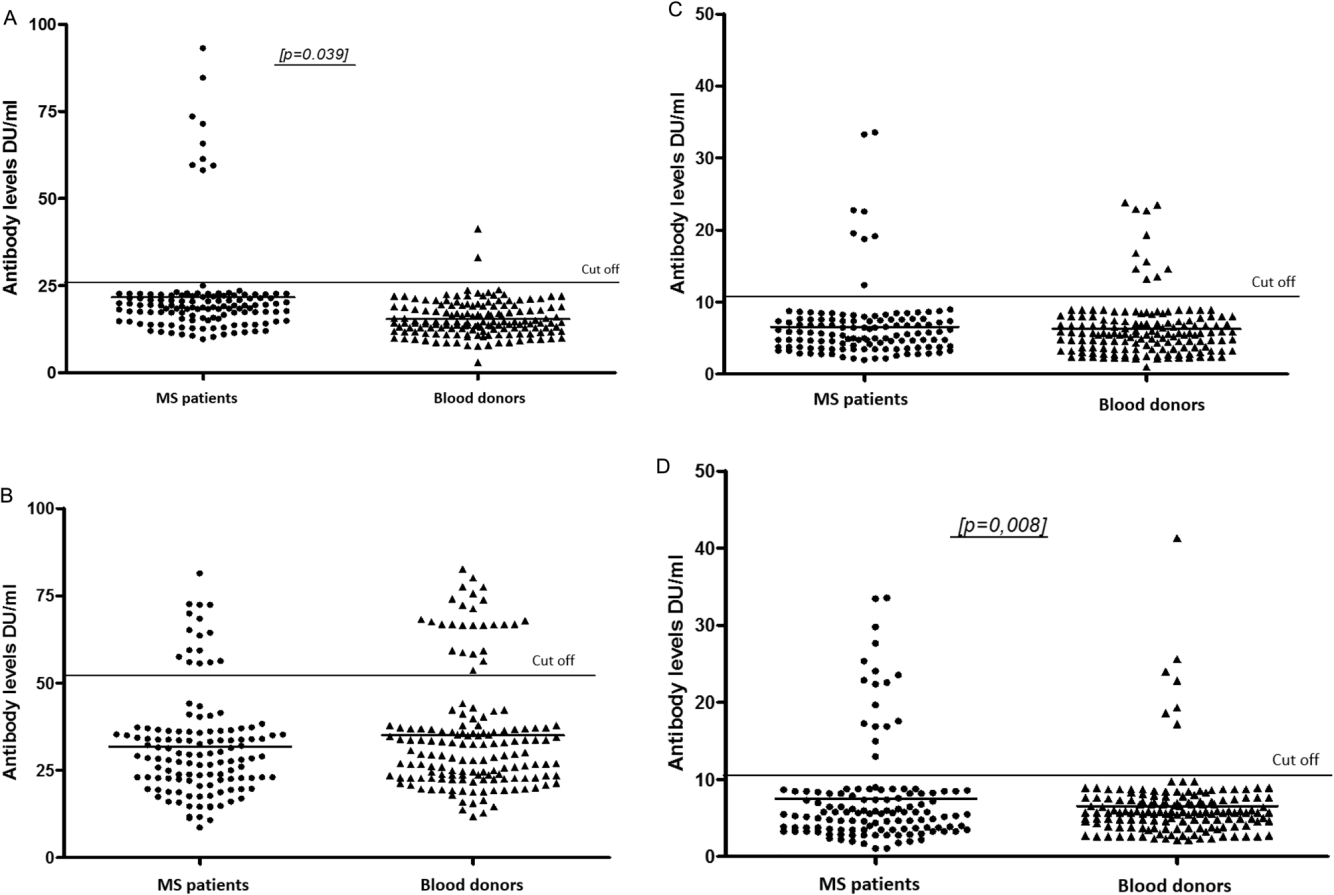
Anti-*T. gondii* IgM (**A**) and IgG (**B**) and anti-*B. burgdorferi* IgM (**C**) and IgG (**D**) levels in the serum samples collected from MS patients and blood donors.

The IgM seroprevalence was about 6 times higher in MS patients (7.3%; 9/124; 95% CL 3.7–12.8%) than in the blood of healthy donors (1.3%; 2/150; 95% CL 0.3–4.2%) (χ^2^_2_ = 6.49, p = 0.039) (Table 1; Fig. 1A). The IgG seroprevalence did not differ statistically between MS patients and the control group (12.9% [16/124; 95% CL 7.9–19.6%] vs. 17.3% [26/150; 95% CL 11.9–24.0%]; p = 0.712) (Fig. 1B). A significant difference in IgM seroprevalence between MS patients who were treated with IMDs, and those who were not was observed. All IgM-positive serum samples from MS patients (n = 9) were obtained from individuals with IMD treatment (interferon beta n = 5; dimethyl fumarate n = 2; glatiramer acetate n = 1; teriflunomide n = 1) whereas no specific IgM antibodies in non-treated patients were noted (χ^2^_1_ = 4.04, p = 0.044). No significant association was demonstrated between the serological status of the tested group of MS patients or healthy donors and age (Table 1). All anti-*T. gondii* IgG-positive samples were tested for antibody avidity with equivocal (45–60%; n = 7) or high (> 60%; n = 9) values.

**Table 1:** Stratification by age in cases and controls for IgM and IgG seropositivity to *T. gondii* and *B. burgdorferi*.

Age	Number of tested sample	IgM % (positive) [95% CL]		P value	IgG % (positive) [95% CL]		P value
*Toxoplasma gondii*							
MS patients	124	7.3 (9)	[3.7–12.8]	**0.039**	12.9 (16)	[7.9–19.6]	0.712
Blood donors	150	1.3 (2)	[0.3–4.2]		17.3 (26)	[11.9–24.0]	
MS patients				0.181			0.579
< 20	3	0 (0)	na		0 (0)	na	
21–35	37	2.7 (1)	[0.3–11.9]		10.8 (4)	[3.8–23.7]	
36–50	67	9.0 (6)	[3.8–17.5]		14.9 (10)	[7.9–24.9]	
> 51	14	14.3 (2)	[3.1–38.5]		14.3 (2)	[3.1–38.5]	
Blood donors							
< 20	7	14.3 (1)	[1.6–50.1]		28.6 (2)	[6.5–64.8]	
21–35	86	1.2 (1)	[0.1–5.3]		14.0 (12)	[7.9–22.4]	
36–50	42	0 (0)	na		16.7 (7)	[7.8–30.0]	
> 51	14	0 (0)	na		28.6 (4)	[10.5–54.5]	
*Borrelia burgdorferi* s.l							
MS patients	124	6.5 (8)	[3.1–11.8]	0.774	13.8 (17)	[8.6–20.7]	**0.008**
Blood donors	150	7.3 (11)	[4.0–12.3]		4.7 (7)	[2.1–8.9]	
MS patients				0.063			0.215
< 20	3	0 (0)	na		0 (0)	na	
21–35	37	5.4 (2)	[1.1–16.2]		8.1 (3)	[2.3–20.1]	
36–50	67	9.0 (6)	[3.8–17.5]		19.4 (13)	[11.3–30.0]	
> 51	14	0 (0)	na		7.1 (1)	[0.8–28.8]	
Blood donors							
< 20	7	0 (0)	na		0 (0)	na	
21–35	86	5.8 (5)	[2.3–12.3]		4.7 (4)	[1.6–10.7]	
36–50	42	4.8 (2)	[1.0–14.4]		2.4 (1)	[0.3–10.6]	
> 51	14	28.6 (4)	[10.5–54.5]		14.3 (2)	[3.1–38.5]	
Doubtful results of *B. burgdorferi* ELISA test							
MS patients	124	0.8 (1)	na	na	3.2 (4)	na	na
Blood donors	150	0 (0)	na		0 (0)	na	

Significant values are in bold.

*na* not applicable.

### Borrelia burgdorferi s.l. IgM and IgG seroprevalence

Only positive results of ELISA tests confirmed by Western Blot were analysed. Among 124 serum samples collected from MS patients, 8 (6.5%; 95% CL 3.1–11.8%) for IgM and 17 (13.8%; 95% CL 8.6–20.7%) for IgG were positive, respectively (Fig. 1C,D). Of the 150 blood donors in the control group tested for specific IgM and IgG by ELISA test, 11 and 7 were positive, respectively, which corresponds to the seroprevalence rate of 7.3% (95% CL 4.0–12.3%) for IgM and 4.7% (95% CL 2.1–8.9%) for IgG (Table 1; Fig. 1C,D). The IgM seroprevalence noted in both groups did not differ statistically, nevertheless, the IgG seroprevalence observed in MS patients was significantly higher compared to seroprevalence observed in blood donors (13.7% vs. 4.7%; χ^2^_1_ = 7.03, p = 0.008). No significant association was demonstrated between the serological status of the tested group of MS patients/healthy donors and age (Table 1).

### Inconclusive results

The doubtful results of the ELISA test were observed only for *B. burgdorferi* s.l. In MS patients the doubtful results were 0.8% (1/124) and 3.2% (4/124) for IgM and IgG tests, respectively, whereas the doubtful results for this pathogen were not noted in blood donors. All doubtful results were noted in MS patients with the IMDs treatment. Nevertheless, the differences mentioned above between treated and non-treated patients were not statistically significant.

## Discussion

To our knowledge, this is one of the first study on *T. gondii* and *B. burgdorferi* s.l. seroprevalence in MS patients in Poland. The association between toxoplasmosis and MS is still unclear ^13–19^. In an animal model of experimental autoimmune encephalomyelitis (EAE), which is the most commonly used rodent’s model of MS, chronic *T. gondii* infection modulates the immunological response towards the production of anti-inflammatory cytokines (i.e. interleukin-10; ^34^) and prevents blood–brain barrier (BBB) disruption caused by EAE ^35^ what seem to support the hypothesis about the protective effect of *Toxoplasma* infection on the development of autoimmune disorders. We have demonstrated a lower *T. gondii* IgG seropositivity in MS patients (12.9%) compared to control (17.3%) which may further support the protective role of *Toxoplasma* infection in MS. Nevertheless, the observed difference was not statistically significant. In our study, the high avidity of IgG antibodies has suggested past infection, but the exact time of infection (before or after the development of MS) is impossible to determine. Further clinical and experimental studies are needed to confirm the protective role of *Toxoplasma* infection and understand the mechanism of this phenomenon.

In our study, the significant difference in *T. gondii* IgM seroprevalence between MS patients (7.3%) and control group (1.3%) has been noted. It has been shown that *T. gondii* IgM antibodies can persist beyond 2 years after infection ^36^ and therefore IgM detection could not be the marker of acute, primary infection unless it is found at high titres ^6^. In the current study, the IgM ELISA tests with a sensitivity and specificity reported by the manufacturer of > 98% were used and relatively high titres of IgM were observed in all IgM-positive samples (58–93 DU/ml; values considered positive > 28 DU/ml). Interestingly, the MS patients with IgM-positive and IgG-negative (n = 8) as well as with IgM- and IgG-positive (n = 1) results of ELISA tests were receiving IMD treatment. Patients with MS undergoing immunosuppression or immunomodulatory therapies may be at risk of primary infections, reactivation of latent infections or worsening of asymptomatic chronic infections ^37,38^. Currently, available disease-modifying drugs are primarily designed to suppress pathological CNS inflammation and the clinical response and may result i.e. sequestering autoreactive T cells in lymph nodes away from CNS causing lymphopenia (fingolimod; ^39^), increasing expression and concentration of anti-inflammatory molecules (interferon beta; ^40^), altering immune cell function (glatiramer acetate, dimethyl fumarate; ^41,42^) or reducing in proliferation of activated T and B lymphocytes without causing cell death (teriflunomide; ^43^). However, the immune mechanism induced by approved disease-modifying drugs is not clear, because the pathogenesis of MS remains elusive. Single cases of disseminated cryptococcosis, *Pneumocystis* pneumonia as well as CNS and ocular toxoplasmosis were noted in non-HIV-infected individuals without other causes of secondary immunodeficiency, but with MS and receiving IMDs ^39,44–47^. Regarding to retrospective nature of our studies, we have not been able to correlate the obtained results of serological tests with the medical data and potential, clinical manifestation of *Toxoplasma* infection in IgM-positive MS patients. Nevertheless, the risk of opportunistic infections in MS patients must be considered before starting or during the continuation of IMD therapy.

Lyme borreliosis is a multi-system disorder with clinical manifestations involving the patient’s skin, nervous system, joints, or heart and the late, disseminated stage of the disease may mimic the clinical symptoms of MS ^48^. Since uneven distribution of MS worldwide as well as geographical and seasonal corelation of MS to *B. burgdorferi* transmitting *Ixodes* ticks were noted, Fritzsche ^49^ has suggested a causal relation between developing MS and exposure to a spirochaetal virulence factor at conception and birth. Martin and co-authors ^26^ have shown that that molecular mimicry and cross-reactivity between the pathogen and myelin antigens can exacerbate autoimmune diseases in humans. However, whether *B. burgdorferi* (neuro)infection is a cause of MS or MS patients are highly susceptible to *B. burgdorferi* (neuro)infection due to damage of BBB still remains unexplained ^50^.

The *B. burgdorferi* seroprevalence in MS patients was studied previously in the US and Europe and the obtained results were conflicting ^23,24,30,51^. We have confirmed a significant difference in the *B. burgdorferi* IgG seroprevalence between MS patients (13.8%) and control group (4.7%). It seems to indicate the positive correlation between MS and *B. burgdorferi* infections, however, as we have mentioned above, the time of *Borrelia* infection in MS patients (before or after MS development) is impossible to determine. It is worth noting that Poland is believed to be the endemic region for *Ixodes ricinus* ticks as well as for Lyme disease, therefore the positive serology likely reflects previous exposure to *Borrelia* spirochetes rather than active infection. Nevertheless, the associations between *Borrelia* infection and MS need further epidemiological and molecular studies.

In our study, *Borrelia* seroprevalence was estimated based on a two-tiered algorithm (ELISA and Western Blot tests) according to the recommendations of The Polish Society of Epidemiology and Infectious Disease ^32^ and European guidelines ^33^. The frequency of doubtful results for *Borrelia* ELISA tests ranged between 0.8% (IgM) to 3.2% (IgG), whereas no *Borrelia* doubtful results were observed in the control group. All doubtful results were noted in MS patients with IMD treatment. Serological screening tests used in the diagnosis of *B. burgdorferi* infection have a significant potential to generate false positive and equivocal results in patients with immunological disorders and viral infections which may be related to the cross-reactions occurring during polyclonal activation of B cells ^52,53^. A more important problem than lower sensitivity is low test specificity, particularly in the case of IgM antibody ^53^. It cannot be excluded that immunomodulation leading, among others, to lymphocytes B proliferation may be also the cause of not only equivocal results, but also false-positive results, and that the predictive value of diagnostic tests in these cases may be lower ^52^. Diagnostic parameters of individual methods depend on the patient's clinical condition, infectious and non-infectious comorbidities ^53^. This indicates the need for a two-step serological diagnosis of *Borrelia* infection or the use of molecular methods to exclude or confirm infection. There is no conclusive data for patients with MS therefore, results of our study underline the need for this type of research, both in areas where Lyme disease is endemic and in areas where infections are sporadic.

The main limitation of our study was retrospective nature which made impossible to know when *Toxoplasma* or *Borrelia* infections were acquired-before or after the development of MS. Retrospective studies also limited the availability of the information (i) on socioeconomic and educational status of the patients, which could affect *Toxoplasma* seropositivity, as well as (ii) on the clinical manifestation of possibly *Toxoplasma* or *Borrelia* infection in MS patients. The number of tested MS patients and the mean age of population were relatively low. Since the risk of *Toxoplasma* and *Borrelia* seropositivity increase during lifetime ^6,15,16,54^, the high rate of younger people may affect the level in seropositivity and the differences in seropositivity between age classes.

## Conclusions

The results of this study suggest the protective role of *T. gondii* infections in MS patients. High *Toxoplasma* IgM seropositivity in MS patients receiving IMDs therapy was identified. On the other hand, *Borrelia* infections seem to be positively associated with MS. Therefore, in MS patients, prior to continuation of IMDs therapy, serological tests of opportunistic pathogens, including *T. gondii*, should be considered. Nevertheless, the interpretation of our results is limited by the retrospective nature of our studies and what has been discussed. Hence, further experimental and clinical studies are needed to explain the role of infectious agents in the development and pathophysiological mechanisms of MS.

### Supplementary Information

Supplementary Information.

## Data Availability

The datasets used and analysed during this study are available from the corresponding author (RWF) on reasonable request.
