# Supplementary material for: Seroprevalence of Toxoplasma gondii and Borrelia burgdorferi infections in patients with multiple sclerosis in Poland
Source: Sci Rep. 2024 May 14;14:11015. doi: 10.1038/s41598-024-61714-y (PMC11094124; doi:10.1038/s41598-024-61714-y)
Supplement: Supplementary file 1 — Supplementary Information. [file 41598_2024_61714_MOESM1_ESM.docx]

Supplementary File 1. Clinical data of MS patients included to the study

| No of sample | Age | Sex | Type of MS | EDSS | MS duration (years) | Phase of MS (relapse/remission) | Treatment duration (years) | Treatment |
| --- | --- | --- | --- | --- | --- | --- | --- | --- |
| 1 | 31 | M | RRMS | 4 | 5 | remission | nd | Natalizumab |
| 2 | 44 | F | RRMS | 2,5 | 8 | remission | 1 | Interferon beta |
| 3 | 43 | F | RRMS | 2 | 7 | remission | 6 | Interferon beta |
| 4 | 26 | F | RRMS | 1,5 | 1 | remission | nd | Glatiramer acetate |
| 5 | 20 | F | RRMS | 2,5 | 3 | remission | nd | Dimethyl fumarate |
| 6 | 43 | M | RRMS | 2,5 | 3 | remission | nd | Interferon beta |
| 7 | 38 | F | RRMS | 1 | 6 | remission | 6 | Interferon beta |
| 8 | 31 | M | RRMS | 2,5 | 9 | remission | 2 | Interferon beta |
| 9 | 31 | M | RRMS | 2,5 | 6 | remission | nd | Interferon beta |
| 10 | 46 | F | RRMS | 3,5 | 10 | remission | 3 | Glatiramer acetate |
| 11 | 36 | F | RRMS | 3,5 | 15 | remission | nd | Natalizumab |
| 12 | 39 | M | RRMS | 2,5 | 1 | remission | 1 | Interferon beta |
| 13 | 41 | M | RRMS | 2,5 | 1 | remission | 6/12 | Dimethyl fumarate |
| 14 | 39 | M | RRMS | 4 | 5 | remission | 3 | Interferon beta |
| 15 | 45 | M | RRMS | 2,5 | 6 | remission | 6 | Dimethyl fumarate |
| 16 | 56 | F | RRMS | 2,5 | 10 | remission | 7 | Glatiramer acetate |
| 17 | 45 | F | RRMS | 2,5 | 14 | remission | 6 | Interferon beta |
| 18 | 22 | M | RRMS | 1,5 | 3,5 | remission | 3 | Interferon beta |
| 19 | 29 | F | RRMS | 2 | 10 | remission | 6/12 | Dimethyl fumarate |
| 20 | 36 | F | RRMS | 2,5 | 9 | remission | 2 | Teriflunomide |
| 21 | 29 | F | RRMS | 4 | 5 | remission | 6/12 | Dimethyl fumarate |
| 22 | 39 | M | RRMS | 1 | 4 | remission | 3 | Interferon beta |
| 23 | 33 | M | RRMS | 2 | 7 | remission | 6 | Interferon beta |
| 24 | 25 | F | RRMS | 1 | 7 | remission | 5 | Teriflunomide |
| 25 | 33 | F | RRMS | 2 | 15 | remission | 8 | Interferon beta |
| 26 | 31 | F | RRMS | 2 | 7 | remission | 4 | Glatiramer acetate |
| 27 | 47 | F | RRMS | 3 | nd | remission | not treated | na |
| 28 | 32 | F | RRMS | 3 | 15 | remission | nd | Natalizumab |
| 29 | 37 | M | RRMS | 3 | 8 | remission | 5 | Dimethyl fumarate |
| 30 | 27 | F | RRMS | 2,5 | 4/12 | remission | not treated | na |
| 31 | 36 | M | PPMS | 4,5 | nd | na | not treated | na |
| 32 | 34 | F | RRMS | 1,5 | 6 | remission | 1,5 | Dimethyl fumarate |
| 33 | 28 | F | RRMS | 1,5 | 6 | remission | 6 | Glatiramer acetate |
| 34 | 40 | F | RRMS | 1,5 | 5 | remission | not treated | na |
| 35 | 24 | F | RRMS | 3 | 6 | remission | not treated | na |
| 36 | 40 | F | RRMS | 1,5 | 5 | remission | nd | Glatiramer acetate |
| 37 | 22 | M | RRMS | 1,5 | 2 | remission | not treated | na |
| 38 | 40 | F | RRMS | 1 | 1 | remission | not treated | na |
| 39 | 19 | M | RRMS | 2 | 3/12 | remission | not treated | na |
| 40 | 41 | M | RRMS | 3 | 5 | remission | not treated | na |
| 41 | 25 | F | RRMS | 1 | 3/12 | remission | not treated | na |
| 42 | 48 | M | PPMS | 2 | nd | na | not treated | na |
| 43 | 37 | F | RRMS | 3 | 13 | remission | not treated | na |
| 44 | 31 | F | RRMS | 2,5 | 5 | remission | not treated | na |
| 45 | 47 | F | RRMS | 3 | nd | remission | not treated | na |
| 46 | 55 | F | RRMS | 2 | 2 | remission | not treated | na |
| 47 | 23 | F | RRMS | 3 | 1 | remission | not treated | na |
| 48 | 44 | M | RRMS | 5,5 | nd | remission | not treated | na |
| 49 | 44 | M | RRMS | 1,5 | 10 | remission | nd | Interferon beta |
| 50 | 31 | F | RRMS | 0 | 1 | remission | no treated | na |
| 51 | 36 | F | RRMS | 2 | 4 | remission | no treated | na |
| 52 | nd | nd | RRMS | 2 | 3 | remission | no treated | na |
| 53 | nd | nd | RRMS | nd | nd | remission | no treated | na |
| 54 | 32 | F | RRMS | nd | nd | remission | nd | nd |
| 55 | 53 | F | RRMS | 2,5 | 10 | remission | nd | Natalizumab |
| 56 | 48 | F | RRMS | 3 | nd | remission | not treated | na |
| 57 | 33 | F | RRMS | nd | nd | remission | nd | Glatiramer acetate |
| 58 | 30 | F | RRMS | 1,5 | 11 | remission | nd | Natalizumab |
| 59 | 47 | M | RRMS | 5,5 | 8 | remission | nd | Natalizumab |
| 60 | 28 | F | RRMS | 2,5 | 3 | remission | not treated | na |
| 61 | 36 | F | RRMS | 1,5 | 10 | remission | nd | Interferon beta |
| 62 | 53 | F | RRMS | 4,5 | 15 | remission | nd | Natalizumab |
| 63 | 51 | F | RRMS | 2,5 | 12 | remission | nd | Natalizumab |
| 64 | 36 | F | RRMS | 2,5 | 9 | remission | nd | Natalizumab |
| 65 | 33 | M | RRMS | 1,5 | 6 | remission | nd | Natalizumab |
| 66 | 35 | F | RRMS | 1,5 | 15 | remission | nd | Glatiramer acetate |
| 67 | 44 | F | RRMS | nd | 14 | remission | nd | Natalizumab |
| 68 | 40 | M | RRMS | nd | nd | remission | nd | Teriflunomide |
| 69 | 44 | F | RRMS | nd | nd | remission | nd | nd |
| 70 | 38 | M | RRMS | 4 | 10 | remission | nd | Glatiramer acetate |
| 71 | 36 | F | RRMS | 3,5 | 12 | remission | not treated | na |
| 72 | 25 | F | RRMS | nd | nd | remission | nd | nd |
| 73 | 49 | F | RRMS | nd | nd | remission | nd | nd |
| 74 | 38 | M | RRMS | nd | nd | remission | nd | nd |
| 75 | 28 | F | RRMS | 6 | 5 | remission | nd | Glatiramer acetate |
| 76 | 36 | F | RRMS | 3 | 7 | remission | nd | Glatiramer acetate |
| 77 | nd | nd | RRMS | nd | nd | remission | nd | Dimethyl fumarate |
| 78 | 53 | F | RRMS | 1,5 | 8 | remission | nd | Glatiramer acetate |
| 79 | 37 | F | RRMS | 2 | 15 | remission | nd | Glatiramer acetate |
| 80 | 46 | F | RRMS | nd | nd | remission | nd | nd |
| 81 | 24 | F | RRMS | nd | nd | remission | nd | nd |
| 82 | 40 | F | RRMS | 2,0 | nd | remission | nd | na |
| 83 | 42 | F | RRMS | nd | nd | remission | nd | nd |
| 84 | 43 | F | RRMS | 3 | 18 | remission | nd | Glatiramer acetate |
| 85 | 55 | F | RRMS | 4,5 | 14 | remission | nd | Glatiramer acetate |
| 86 | 36 | F | RRMS | nd | nd | remission | nd | Dimethyl fumarate |
| 87 | 36 | F | RRMS | 1,5 | 11 | remission | nd | Glatiramer acetate |
| 88 | 45 | F | RRMS | 3 | 4,5 | remission | nd | Fingolimod |
| 89 | 55 | F | RRMS | nd | nd | remission | nd | Glatiramer acetate |
| 90 | 58 | F | RRMS | 1,5 | 19 | remission | nd | Glatiramer acetate |
| 91 | 36 | M | RRMS | 4,5 | 17 | remission | nd | Glatiramer acetate |
| 92 | 37 | F | RRMS | 2,5 | 13 | remission | nd | Glatiramer acetate |
| 93 | 47 | F | RRMS | 3,5 | 10 | remission | nd | Natalizumab |
| 94 | 39 | F | RRMS | 1,5 | 5 | remission | 5 | Glatiramer acetate |
| 95 | 38 | F | RRMS | 1 | 3 | remission | nd | Interferon beta |
| 96 | 37 | F | RRMS | 0 | 8 | remission | 6 | Interferon beta |
| 97 | 48 | M | RRMS | 1 | 9 | remission | 8 | Glatiramer acetate |
| 98 | 51 | F | RRMS | 1 | 5 | remission | 5 | Interferon beta |
| 99 | 40 | F | RRMS | 1,5 | 6 | remission | 3 | Interferon beta |
| 100 | 40 | F | RRMS | 1,5 | 6 | remission | 3 | Interferon beta |
| 101 | 39 | F | RRMS | 3,5 | 3 | remission | 3 | Teriflunomide |
| 102 | 40 | M | RRMS | 3,5 | 3 | remission | 3 | Teriflunomide |
| 103 | 44 | M | RRMS | 1,5 | 18 | remission | nd | Natalizumab |
| 104 | 40 | F | RRMS | 1 | 10 | remission | 10 | Interferon beta |
| 105 | 43 | F | RRMS | 2 | 4 | remission | 4 | Interferon beta |
| 106 | 52 | M | RRMS | 5,0 | 6 | remission | nd | Interferon beta |
| 107 | 30 | M | RRMS | 2,0 | 4 | remission | nd | Interferon beta |
| 108 | 23 | M | RRMS | 1,0 | 6 | remission | 5 | Dimethyl fumarate |
| 109 | 27 | F | RRMS | 1,5 | 1 | remission | nd | Interferon beta |
| 110 | 42 | F | RRMS | 3,5 | 15 | remission | nd | Natalizumab |
| 111 | 48 | F | RRMS | 1,5 | 19 | remission | 7 | Interferon beta |
| 112 | 42 | F | RRMS | 2,5 | 6 | remission | nd | Dimethyl fumarate |
| 113 | 52 | M | RRMS | 2,0 | 4 | remission | nd | Interferon beta |
| 114 | 36 | M | RRMS | 2,5 | 1 | remission | nd | Dimethyl fumarate |
| 115 | 35 | M | RRMS | 1,5 | 3 | remission | nd | Glatiramer acetate |
| 116 | 31 | M | RRMS | 2,0 | 1 | remission | nd | Glatiramer acetate |
| 117 | 37 | F | RRMS | 5 | 13 | remission | nd | Natalizumab |
| 118 | 48 | M | RRMS | 4,0 | 3 | remission | nd | Dimethyl fumarate |
| 119 | 51 | F | RRMS | 3,0 | 13 | remission | 2 | Interferon beta |
| 120 | 56 | M | RRMS | 3,0 | 5/12 | remission | 5/12 | Glatiramer acetate |
| 121 | 23 | F | RRMS | 2,0 | 1 | remission | 1 | Dimethyl fumarate |
| 122 | 38 | F | RRMS | 2 | 18 | remission | nd | Teriflunomide |
| 123 | 19 | M | RRMS | 3,0 | 1 | remission | 1 | Dimethyl fumarate |
| 124 | 34 | F | RRMS | 2,5 | 3 | remission | 3 | Glatiramer acetate |

na – not applicable

nd – not data
